# Account of Diseases in an Eastern District of London, from the 20th of May to the 20th of June

**Published:** 1799-07

**Authors:** 


### STATE OF DISEASES IN LONDON.

*Account of Diseases in an Eastern District of London, from the 20th of May to the 20th of June.*

| ACUTE DISEASES. | No. of Cases. |
|---|---|
| Peripneumonia Notha | 2 |
| Intermittent Fever | 1 |
| Typhus Mitior | 4 |
| Scarlatina | 1 |
| Ophthalmia | 3 |
| Acute Rheumatifm | 2 |
| **CHRONIC DISEASES.** | |
| Cough | 6 |
| Dyfpnœa | 3 |
| Cough and Dyfpnœa | 7 |
| Phthifis Pulmonalis | 8 |
| Hæmoptoe | 3 |
| Hoarfenefs | 2 |
| Pleurodyne | 3 |
| Hydrothorax | 2 |
| Afcites | 3 |
| Anafarca | 2 |
| Ophthalmia | 1 |
| Cephalalgia | 6 |
| Hemicrania | 1 |
| Paralyfis | 2 |
| Hemiplegia | 1 |
| Epiftaxis | 2 |
| Gaftrodynia | 7 |
| Dyfpepfia | 6 |
| Vomitus | 3 |
| Pyrofis | 1 |
| Enterodynia | 4 |
| Procidentia Vaginæ | 2 |
| Hæmorrhois | 3 |
| Diarrhœa | 2 |
| Obftipatio | 3 |
| Dyfuria | 6 |
| Hyfteria | 4 |
| Hypochondriafis | 3 |
| Palpitation of the Heart | 2 |
| Tremor | 1 |
| Scrofula | 4 |
| Herpes | 4 |
| Tinea | 3 |
| Chronic Rheumatifm | 10 |
| Gout | 2 |
| **PUERPERAL DISEASES.** | |
| Menorrhagia Lochialis | 4 |
| Enurefis | 2 |
| Stranguria | 3 |
| Maftrodynia | 7 |
| **INFANTILE DISEASES.** | |
| Convulfio | 2 |
| Ophthalmia | 3 |
| Aphthæ | 3 |
| Herpetic Eruptions | 4 |

A confiderable change in the ftate of the weather having taken place fince the laft report, the number of difeafes depending upon it has been diminifhed; the wind, however, ftill continuing to blow from the Eaft and North-eaft, complaints of the cheft ftill continue to harrafs many patients, though the number of recent inftances is much fmaller.

Cafes of ophthalmia, a difeafe particularly noticed in the laft month, are ftill numerous; owing, probably, to the ftate of the weather juft referred to.

An inftance of pyrofis having prefented itfelf, and it being a difeafe of rare occurrence, we embrace the opportunity of taking fome notice of it. This difeafe, though in fome of its fymptoms it bears a near refemblance to other morbid affections of the ftomach, as dyfpepfia, gaftrodynia, cardialgia, is particularly characterized by the frequent eructation of a watery, infipid fluid, on which account it is diftinguifhed in Scotland by the name of water brafh.

braſh. This eructation is generally preceded by pain in the region of the organ, accompanied with a ſenſe of ſtricture, which has occaſioned its being ranked by noſologiſts amongſt ſpaſmodic diſeaſes. This complaint in ſome inſtances returns periodically, and generally in the morning or forenoon, when the ſtomach is empty. The patient complains of pain, attended with a ſenſe of heat, ſimilar to what is called the heart-burn; the ſtomach is ſuddenly provoked to throw up its contents, and a thin watery fluid is diſcharged.

It has been obſerved, that this diſeaſe more frequently attacks the female than the male, and that a ſtate of pregnancy renders the patient more liable to the attack. It is commonly found amongſt the lower claſſes of ſociety, and has been attributed to a farinaceous diet. In the inſtance referred to in the liſt, there were ſymptoms of too liberal a uſe of ſpirituous liquors.

As the pathology of the diſeaſe is not very well underſtood, ſo the propereſt mode of treatment has not been aſcertained.

The ſymptoms are palliated by the uſe of opium, conſiderable effects have been attributed to the nux vomica; and it has been aſſerted, that the chewing of tobacco has proved beneficial.

